# Medicaid expansion and opioid supply policies to address the opioid overdose crisis

**DOI:** 10.1016/j.dadr.2022.100042

**Published:** 2022-03-20

**Authors:** Shishir Shakya, Samantha J. Harris

**Affiliations:** a Department of Economics, Shippensburg University of Pennsylvania, USA; b Department of Health Policy and Management, Johns Hopkins Bloomberg School of Public Health, USA

**Keywords:** Buprenorphine, Opioid use disorder, Medicaid, Pain management clinic

## Abstract

*Background:* The opioid overdose crisis remains of critical concern after historic increases in overdose mortality in the United States between 2020 and 2021. Improving access to buprenorphine —a partial opioid agonist and one of three FDA-approved medications for opioid use disorder (OUD) treatment— and reducing inappropriate opioid prescriptions may help curb mortality. Here, we examined the impact of Medicaid expansion and pain management clinic laws on opioid prescription rates and buprenorphine availability. *Methods:* We examined both retail opioid prescriptions per 100 persons in the state population using data from the Centers for Disease Control and Prevention and data on buprenorphine distributions in kilograms per 100,000 persons in the state population from the Automated Reports and Consolidated Ordering System database. We employed difference-in-difference frameworks to estimate the impact of Medicaid expansion on buprenorphine access and retail opioid prescription rates. Models considered three separate treatment variables: Medicaid expansion, pain management clinic (“pill mill”) laws, and the interaction of Medicaid expansion and pain management clinic laws. *Results:* Findings showed that Medicaid expansion was associated with increased access to buprenorphine in expansion states that also employed more stringent supply-side policies, including pain management clinic laws, relative to states that did not implement policies targeting the over-supply of prescription opioids over the same time period. Conclusions. Together, Medicaid expansion and policies limiting inappropriate opioid prescriptions show promise for improving the accessibility of buprenorphine treatment for OUD.

## Highlights

• The opioid overdose crisis remains a chief public health concern in the United States.
• Buprenorphine availability for opioid use disorder treatment remains limited.
• Medicaid expansion states with pain management clinic (pill mill) laws had higher treatment availability.
• Limiting opioid supply while improving buprenorphine access is a promising strategy.

## 1 Introduction

Many nations have seen increases in opioid involved overdose deaths in recent years, with opioids accounting for over 70 percent of drug-involved deaths worldwide (WHO, 2020). The United States has seen disproportionately high increases in fatal opioid overdoses, with a record 74,000 opioid overdose deaths between 2020 and 2021 (CDC, 2020). In 2018, an estimated 10.1 million Americans misused opioids (NSDUH, 2019). The overdose crisis can be attributed in part to the over-supply of prescription drugs (Pacula and Powell, 2018) and over-prescribing (Manchikanti et al., 2012), changes in the drug supply, high rates of chronic pain and co-morbidities, and socio-economic conditions (Dasgupta et al., 2018;Ruhm, 2019). In 2018, of the 1.6 million Americans that met diagnostic criteria for OUD, only 18 percent received one of the three FDA-approved medications– buprenorphine, injectable naltrexone, or methadone– for OUD treatment (NSDUH, 2019). Furthermore, access to evidence-based medication treatment remains low and is increasingly concerning amid the COVID-19 pandemic (Alexander et al., 2020).

The Affordable Care Act of 2010 expanded Medicaid coverage for millions of Americans. In 2014, the Mental Health Parity and Addiction Equity Act intended to ensure equal access to mental health and substance use disorder treatment services for the Medicaid population, a population with high demand for these services. Prior studies have found improved access to for substance use disorder in Medicaid expansion states (Andrews, Pollack, Abraham, Grogan, Bersamira, D’Aunno, Friedmann, 2019, Maclean, Saloner, 2019, Meinhofer, Witman, 2018, Saloner, Levin, Chang, Jones, Alexander, 2018, Sharp, Jones, Sherwood, Kutsa, Honermann, Millett, 2018, Wen, Hockenberry, Borders, Druss, 2017). However, opioid policies often are interlacing and it can be challenging to measure their impact (Schuler et al., 2020).

Various supply-side policies such as pain management clinic laws and mandatory Prescription Drug Monitoring Programs (PDMPs)restrict the supply of prescription opioids. For example, pain management clinic laws impose strict regulations on opioid prescriptions without medical indication (Brighthaupt et al., 2019). Such supply-side restrictions may reduce opioid mortality (Buchmueller and Carey, 2018) but can cause unintended consequences such as a substitution of prescription opioids with illicit opioids like heroin and fentanyl, and thus increase the risk of overdose or the onset of opioid use disorder (Grecu et al., 2019). Meanwhile, demand-side policies such as Medicaid expansion, Good Samaritan laws, naloxone access laws, and harm-reduction methods aim to meet the demand for treatment and/or reduce overdose.

A recent review of the opioid policy literature by Schuler et al. (2020) found that few studies have examined the relationship between buprenorphine access and pain management clinic laws. Our paper aims to further the literature on the impact of Medicaid expansion and opioid supply (pain management clinic laws) on the retail opioid prescription rate and availability of buprenorphine. Prior studies examining the effects of pain management clinic regulations on the overdose crisis have mainly focused on single states (e.g., Chang, Lyapustina, Rutkow, Daubresse, Richey, Faul, Stuart, Alexander, 2016, Chang, Murimi, Faul, Rutkow, Alexander, 2018, Kennedy-Hendricks, Richey, McGinty, Stuart, Barry, Webster, 2016, Rutkow, Vernick, Alexander, 2017). Dowell et al. (2016) examined the impact of pain management clinic laws across multiple states and found that must-access PDMPs and pain management clinic laws were associated with reduced opioid prescribing and opioid overdose deaths. The current study is the first to examine the impact of Medicaid expansion and pain management clinic laws on opioid prescription rates and buprenorphine availability.

Based on the above literature, our conceptual framework considered that at first, a restrictive supply-side policy like pain management clinic laws could prevent new OUD diagnoses from occurring by limiting over-prescribing. Second, Medicaid expansion has been shown to improve the accessibility of buprenorphine treatment for OUD (Meinhofer, Witman, 2018, Saloner, Levin, Chang, Jones, Alexander, 2018, Sharp, Jones, Sherwood, Kutsa, Honermann, Millett, 2018, Wen, Hockenberry, Borders, Druss, 2017). Last, supply and access policies taken together could create a balance between preventing new-onset of OUD through inappropriate opioid prescribing while ensuring access to quality treatment for those who currently have or develop OUD in the future.

## 2 Methods

### 2.1 Data

Following several studies (e.g., Hudnall, Yang, Melnykov, Zhu, Lewis, Parton, 2022, Pashmineh Azar, Cruz-Mullane, Podd, Lam, Kaleem, Lockard, Mandel, Chung, Simoyan, Davis, et al., 2020, Sairam Atluri, Manchikanti, 2014), we used the Automated Reports and Consolidated Ordering System (ARCOS) database to compile state-level retail buprenorphine distributions from 2006 to 2017. The ARCOS database contains manufacturer and distributor controlled substances transactions data reported to the Drug Enforcement Administration (DEA). Along with ARCOS, we retrieved CDC estimates of state-level retail opioid prescribing rates from 2006 to 2017. The CDC estimates were drawn from the IQVIA Xponent data set and estimated retail opioid prescribing rates. We identified states that expanded Medicaid using Kaiser Family Foundation data, and we identified states that enacted pain management clinic laws from Rutkow et al. (2017).

We compiled information regarding PDMP operations, Good Samaritan laws, and naloxone access laws across states from the Prescription Drug Abuse Policy System website. We also gathered data on if a state has some form of marijuana legalization (medical or/and recreational possession of marijuana) from ProCon’s website. Data on the state-level population, poverty rate, unemployment rate, log of Gross State Product in 2012 constant dollars, and Medicaid Beneficiary population data were drawn from the University of Kentucky Center for Poverty Research (2019). Finally, we deflated the Gross State Product using the US Bureau of Economic Analysis’s implicit price deflator of the 2012 constant dollar. This study was determined as exempt from the Shippensburg University of Pennsylvania Institutional Review Board.

#### 2.1.1 Outcome variables

We considered two separate dependent variables in our study. First, we examined retail opioid prescriptions dispensed per 100 persons in the state population. Second, we examined buprenorphine distributions in kilograms per 100,000 persons in the state population.

#### 2.1.2 Treatment variables

We considered three separate treatment variables: Medicaid expansion, pain management clinic laws, and the interaction of Medicaid expansion and pain management clinic laws. We defined Medicaid expansion as a policy that increased access to health services and medications. In contrast, pain management clinic laws are a supply-side policy that the over-utilization and over-prescribing of opioids. A mix of these two policies could strike a balance between limiting the oversupply of prescription opioids and improving access to OUD treatment.

#### 2.1.3 Covariates

We considered covariates as binary indicator variables of “must-access” PDMP laws, Good Samaritan laws, naloxone access laws, and some form of marijuana legalization (medical or/and recreational possession of marijuana) along with continuous variables such as poverty rate, unemployment rate, and log of Gross State Product in 2012 constant dollars.

We identified PDMP implementation dates in each state and differentiated between states requiring prescribers to consult the state’s PDMP before prescribing controlled substances (the essence of a must-access PDMP). We also included covariates for Good Samaritan laws and naloxone access laws. States with Good Samaritan laws provide immunity from prosecution to individuals possessing a controlled substance while seeking help for themselves or another person experiencing an overdose. States with naloxone access laws often provide naloxone and other opioid overdose prevention services to individuals who use drugs, their friends and family, as well as to first responders. Prevention services include education about overdose risk factors, identifying and appropriately responding to an overdose, and administering naloxone– an opioid overdose reversal medication. As of 2016, 37 states had implemented a Good Samaritan law and 48 states had implemented some variant of naloxone access laws (Ayres and Jalal, 2018).

### 2.2 Statistical analysis

We exploited variation in the timing of adopting various relevant policies within generalized difference-in-differences frameworks. A general description of the difference-in-differences framework is given as:

(1) $$Y_{it}=\alpha +\delta D_{it}+X_{it}\beta +\gamma_{i}+\varsigma_{t}+\omega_{i}t+\varepsilon_{it}$$

where i and t index for state and year. In the above regression, $Y_{it}$ is our main dependent variable. We considered dependent variables as retail opioid prescriptions dispensed per 100 persons in the state population and buprenorphine distributions in kilograms per 100,000 in the state population. $D_{it}$ are various relevant policy treatment variables. As explained earlier, we defined Medicaid expansion, pain management clinic laws, and their interaction as the treatment variables. $X_{it}$ is a matrix that comprised a vector of various control variables or covariates.

$\gamma_{i}$ represents state fixed effects which allowed controlling for any state-specific stable unmeasured heterogeneity. For example, state fixed effects can capture secular differences resulting from unmeasured cultural factors, physician practice styles specific to states, or steady demand of opioids (Grecu et al., 2019). $\varsigma_{t}$ represents year fixed effects of absorbing unobserved trends in the independent variables and related factors common to the entire population, such as changes in federal-level healthcare policies, overall economic conditions, and temporal changes of opioid demand (Grecu et al., 2019). We also included state-specific linear trend $\omega_{i}t$ to control for differential trends in outcome variables that depend upon states having adopted Medicaid expansion or pain management clinic laws.

We weighted the regression estimates with the state-level Medicaid beneficiary population. Furthermore, standard errors were clustered at the state level to allow for an arbitrary autocorrelation process within states (Bertrand et al., 2004). Finally, statistical significances were generated using heteroskedasticity robust standard errors.

## 3 Results

### 3.1 Choropleth maps

Our study had two dependent variables: retail opioid prescriptions dispensed per 100 persons in the state population, and buprenorphine distributions in kilograms per 100,000 persons in the state population.

#### 3.1.1 Choropleth map of retail opioid prescriptions rate

**Fig. 1:**
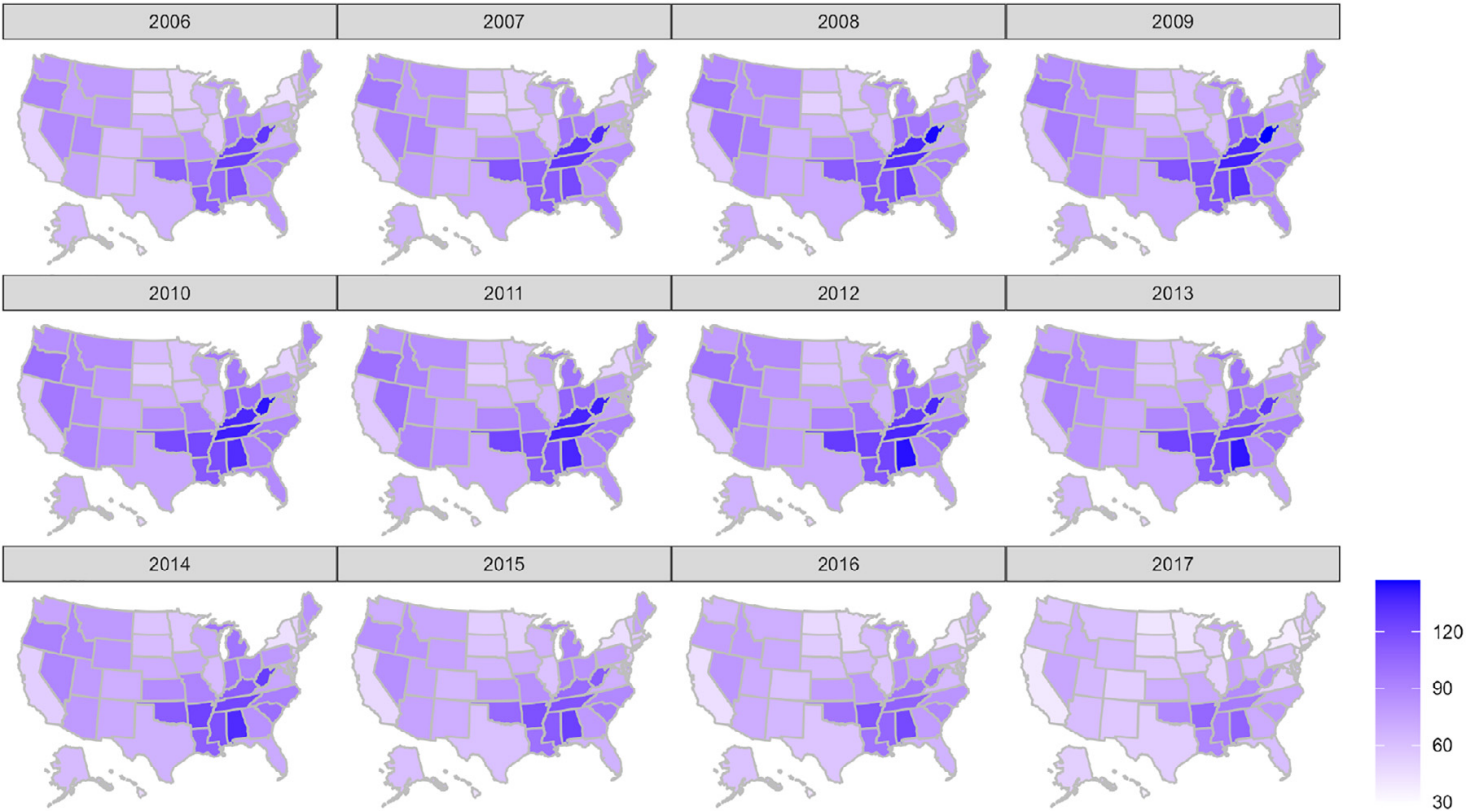
Retail opioid prescriptions dispensed per 100 persons, 2006–2017. *Notes:* The analysis of retail opioid prescriptions dispensed per 100 personsfrom 2006 to 2017 is based on publicly available data from the Centers for Disease Control and Prevention.

Figure 1 exhibits a choropleth map of retail opioid prescriptions dispensed per 100 persons in the state population from 2006 to 2017. Alabama, West Virginia, Tennessee, Kentucky, Arkansas, Mississippi, Oklahoma, and Louisiana were the top 8 states with the highest prescribing rates. In our study, retail opioid prescriptions dispensed per 100 persons proxied opioid prescribing behavior.

#### 3.1.2 Choropleth map of buprenorphine distributions

**Fig. 2:**
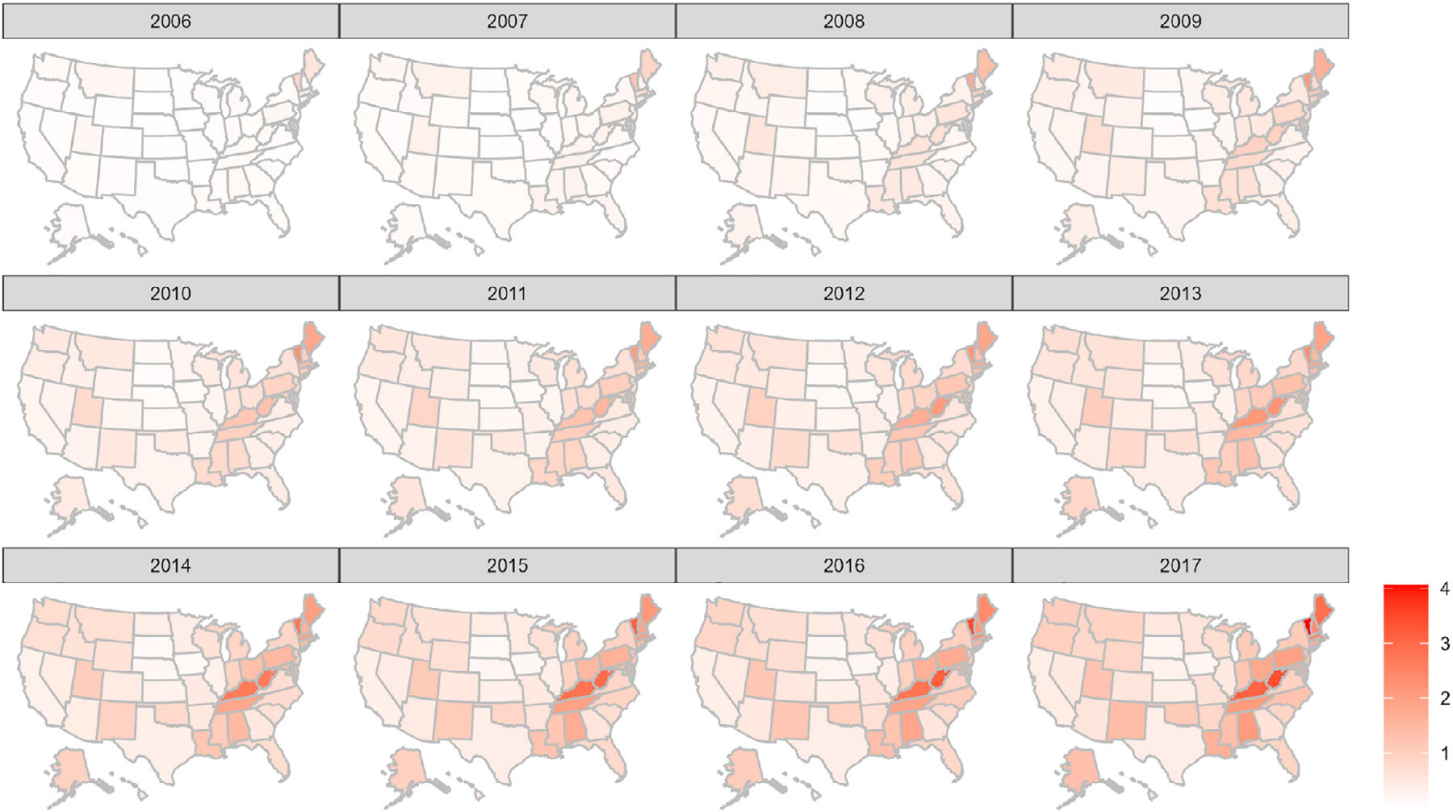
Trends of buprenorphine distributions in kilograms per 100,000 population, 2006–2017. *Notes:* The analysis of buprenorphine distributions in kilograms per 100,000 in the population from 2006 to 2017 is based on publicly available data from the Automated Reports and Consolidated Ordering System (ARCOS).

Figure 2 displays a choropleth map of trends of buprenorphine distributions in kilograms per 100,000 in the state population from 2006 to 2017. Vermont, West Virginia, Maine, Rhode Island, Kentucky, and Massachusetts had some of the highest buprenorphine distributions in kilograms per 100,000 in the population. In our study, buprenorphine distributions in kilograms proxied OUD treatment medication access.

#### 3.1.3 Choropleth map of Medicaid expansion status

**Fig. 3:**
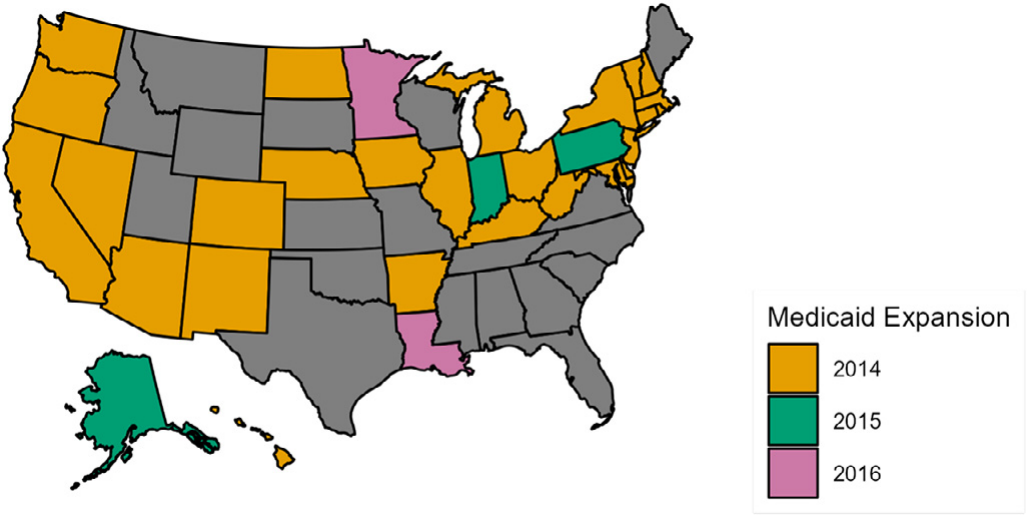
Medicaid expansion. *Notes:* The analysis of Medicaid expansion effective dates is based on publicly available data from Kaiser Family Foundation. The color codes show the effective dates of coverage following Medicaid expansion.

Figure 3 depicts Medicaid expansion status across states. While many states expanded Medicaid in 2014, Alaska, Indiana, and Pennsylvania expanded Medicaid in 2015, and Louisiana and Minnesota did not expand Medicaid until 2016.

#### 3.1.4 Choropleth map of pain management clinic laws

**Fig. 4:**
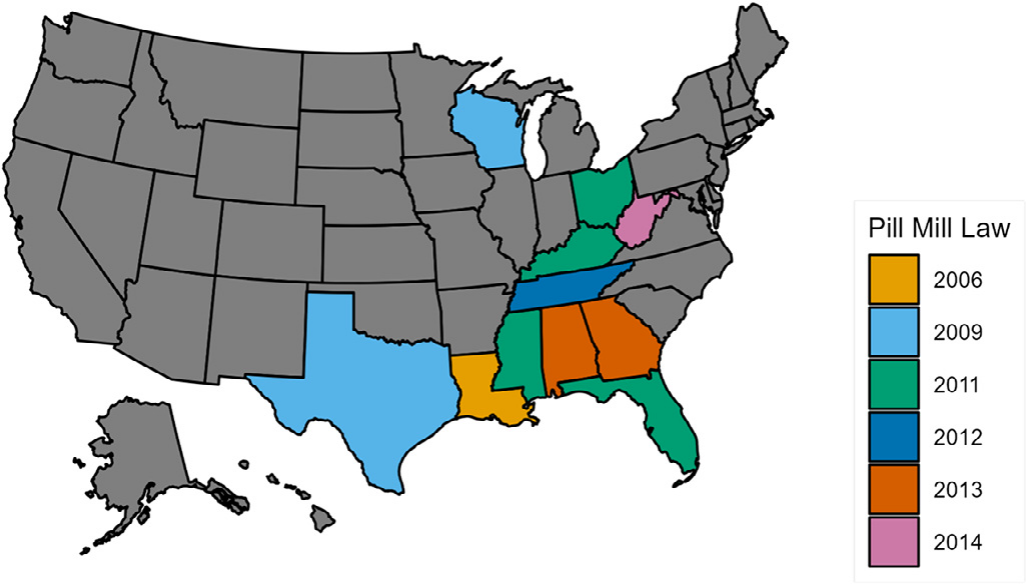
Pain management clinic (“pill mill”) laws. *Notes:* The analysis of state oversight of pain management clinics is based on publicly available data from Rutkow et al. (2017). State law defines what constitutes a pain management clinic, with definitions falling into one or more of three categories: (1) providing pain management services with prescription controlled substances; (2) advertising pain management services; and (3) prescribing controlled substances forpain to a majority of patients Rutkow et al. (2017). The color codes show the effective dates.

Figure 4 shows states that enacted pain management clinic laws during our observation period. Pain management clinic laws are a supply-side restriction policy that targets clinics with a disproportionately high volume of controlled substance prescriptions, including opioids. These laws regulate pain management clinics via increased state oversight by setting a legal definition for what constitutes a clinic in each state. These laws then impose additional regulatory requirements such as routine inspections, dispensing, and clinic ownership limitations, as well as civil and criminal penalties for violations of state laws (Rutkow et al., 2017). Louisiana implemented pain management clinic laws in 2006; Texas and Wisconsin in 2009; Ohio, Mississippi, Kentucky, and Florida implemented in 2011; Tennessee in 2012; Georgia and Alabama in 2013; and West Virginia in 2014.

With Figs. 3 and 4, we show that Kentucky, Ohio, and West Virginia had a mix of Medicaid expansion and pain management clinics laws after 2014, while Louisiana had such policy intersection only after 2016. Hence, four states in our study enacted pain management clinic laws and expanded Medicaid.

### 3.2 Difference-in-differences

In the following section, we examine to what extent Medicaid expansion, pain management clinic laws, and the interaction of these two laws impacted the retail opioid prescription rate using a difference-in-differences framework.

#### 3.2.1 Impacts of Medicaid and pill mill law on retail opioid prescriptions

Table 1 shows the effects of Medicaid expansion, pain management clinic laws, and their interaction on retail opioid prescriptions.

**Table 1:** Impacts of Medicaid and pain management clinic laws on retail opioid prescriptions.

	Retail opioid prescriptions dispensed per 100 persons					
	(1)	(2)	(3)	(4)	(5)	(6)
Medicaid	1.719${}^{**}$		1.432${}^{*}$	1.196		2.253${}^{**}$
	(0.857)		(0.788)	(1.015)		(1.082)
Painmanagementclinic		$-6.552^{***}$	$-4.926^{***}$		$-2.699^{**}$	$-3.136^{**}$
		(0.932)	(0.938)		(1.344)	(1.291)
Painmanagementclinic×Medicaid			$-9.220^{***}$			$-4.982^{**}$
			(1.967)			(2.149)
State fixed effects	Yes	Yes	Yes	Yes	Yes	Yes
Year fixed effects	Yes	Yes	Yes	Yes	Yes	Yes
State specific trends	No	No	No	Linear	Linear	Linear
Controls	Yes	Yes	Yes	Yes	Yes	Yes
Cluster SE	State	State	State	State	State	State
HAC SE	Yes	Yes	Yes	Yes	Yes	Yes
Observations	612	612	612	612	612	612
$R^{2}$	0.975	0.978	0.980	0.989	0.990	0.990
Adjusted $R^{2}$	0.972	0.975	0.978	0.987	0.987	0.987

*Notes:* The analysis of retail opioid prescriptions dispensed per 100 persons is based on publicly available data from the Centers for Disease Control and Prevention. Data on state oversight of pain management clinics is publicly available from Rutkow et al. (2017). Data on state Medicaid expansion status was derived from publicly available data from the Kaiser Family Foundation. Controls include: “must-access” PDMP, Good Samaritan laws, and naloxone access laws available from the Prescription Drug Abuse Policy System’s website; some form of marijuana legalization (medical or/and recreational possession of marijuana) from ProCon's website; poverty rate, unemployment rate, log of Gross State Product in 2012 constant dollars, and Medicaid beneficiary population were drawn from the University of Kentucky Center for Poverty Research’s National Welfare Data. Gross State Product is deflated to 2012 dollars using implicit price deflator of the 2012 constant dollar based on publicly available data from the Bureau of Economic Analysis. Data is a balanced panel for 50 U.S. states and the District of Columbia from 2006 to 2017 and comprises 612 observations. Standard errors are clustered at the state-level and robust to heteroskedasticity. Estimates are weighted with the state-level Medicaid beneficiary population. *** p<0.01, ** p<0.05, * p<0.1.

Table 1 column (1) shows that states that expanded Medicaid had 1.719 (p<.05) more retail opioid prescriptions dispensed per 100 persons in the state population compared to states that did not expand Medicaid. This result exhibits that Medicaid is moderately associated with increased access to prescription opioids. Column (2) shows that states with pain management clinic laws have 6.552 (p<.01) fewer retail opioid prescriptions dispensed per 100 persons relative to states without pain management clinic laws. However, taken together, column (3) shows a substantial reduction of 9.22 retail opioids dispensed per 100 persons (p<.01) in states with both Medicaid expansion and pain management clinic laws in effect.

In Table 1 columns (4) to (6), we account for state-specific linear trends which control for differential trends in the independent variables. Column (4) shows that Medicaid expansion states did not see statistically significant changes in retail opioid prescriptions. In contrast, column (5) shows that states with pain management clinic laws had a reduction of 2.699 (p<.05) retail opioid prescriptions, and column (6) shows states with both of these laws have a further reduction of 4.982 (p<.05) retail opioid prescriptions dispensed per 100 persons in the state population.

In terms of magnitude, results from Table 1 column (1)–(6) suggest that a policy mix of compassionate care policies (e.g., Medicaid expansion and thus, improved access to buprenorphine) and restrictive policies (e.g., pain management clinic regulations) could be well suited to combat the overdose crisis than policies targeting supply or demand alone.

#### 3.2.2 Impacts of Medicaid and pill mill law on buprenorphine distributions

Table 2 provides the impact of Medicaid expansion, pain management clinic laws, and their interaction on buprenorphine distributions.

**Table 2:** Impacts of Medicaid and pain management clinic laws on buprenorphine distributions.

	Buprenorphine distributions, kilograms per 100,000 persons					
	(1)	(2)	(3)	(4)	(5)	(6)
Medicaid	$-0.066^{*}$		$-0.092^{**}$	−0.019		$-0.051^{***}$
	(0.038)		(0.036)	(0.014)		(0.014)
Painmanagementclinic		0.197${}^{***}$	0.106${}^{**}$		0.019	0.031${}^{*}$
		(0.048)	(0.047)		(0.020)	(0.018)
Painmanagementclinic×Medicaid			0.494${}^{***}$			0.189${}^{***}$
			(0.099)			(0.048)
State fixed effects	Yes	Yes	Yes	Yes	Yes	Yes
Year fixed effects	Yes	Yes	Yes	Yes	Yes	Yes
State specific trends	No	No	No	Linear	Linear	Linear
Controls	Yes	Yes	Yes	Yes	Yes	Yes
Cluster SE	State	State	State	State	State	State
HAC SE	Yes	Yes	Yes	Yes	Yes	Yes
Observations	612	612	612	612	612	612
$R^{2}$	0.904	0.908	0.919	0.993	0.993	0.994
Adjusted $R^{2}$	0.891	0.897	0.908	0.991	0.991	0.992

*Notes:* The analysis of buprenorphine distributions in kilograms per 100,000 is based on publicly available data from the Automated Reports and Consolidated Ordering System (ARCOS), available from the U.S. Department of Justice, Drug Enforcement Administration, Diversion Control Division. State population data were drawn from the University of Kentucky Center for Poverty Research’s National Welfare Data. Data on state Medicaid expansion status was derived from publicly available data from the Kaiser Family Foundation. Controls include: “must-access” PDMP, Good Samaritan laws, and naloxone access laws available from the Prescription Drug Abuse Policy System’s website; some form of marijuana legalization (medical or/and recreational possession of marijuana) from ProCon's website; poverty rate, unemployment rate, log of Gross State Product in 2012 constant dollars, and Medicaid beneficiary population were drawn from the University of Kentucky Center for Poverty Research’s National Welfare Data. Gross State Product is deflated to 2012 dollars using implicit price deflator of the 2012 constant dollar based on publicly available data from the Bureau of Economic Analysis. Data is a balanced panel for 50 U.S. states and the District of Columbia from 2006 to 2017 and comprises 612 observations. Standard errors are clustered at the state-level and robust to heteroskedasticity. Estimates are weighted with the state-level Medicaid beneficiary population. *** p<0.01, ** p<0.05, * p<0.1.

Table 2 column (1) shows that states that expanded Medicaid had 0.066 (p<.10) less buprenorphine distributions per 100,000 population compared to states that did not expand Medicaid. This estimate is counter-intuitive and also is weak in statistical significance. Column (2) shows that states with pain management clinic laws have 0.197 (p<.01) higher buprenorphine distributions per 100,000 in the state population. While in column (3), the interaction of these two policies yielded 0.494 (p<.01) kilograms higher distributions of buprenorphine per 100,000 population.

Table 2 columns (4) to (6) account for state-specific linear trends. Column (4) shows that buprenorphine distributions per 100,000 in the state population were not statistically significant and provide evidence that the counter-intuitive finding in column (1) is probably invalid. In contrast, column (5) shows that states with pain management clinic laws had a reduction of 2.699 (p<.05) retail opioid prescriptions, and column (6) shows states with both of these laws had a further reduction of 4.982 (p<.05) retail opioid prescriptions dispensed per 100 persons.

Similar to results from Table 1 column (1)–(6), the results from Table 2 column (1)–(6) also suggest a mix of compassionate and restrictive policies is a well-suited strategy for states to improve the availability of OUD treatment.

### 3.3 Robustness checks

Furthermore, as a robustness check we present results without including covariates in Appendix A, Tables A.1, Table A.1 and A.2. The estimates remained similar to Tables 1 and 2, respectively.

## 4 Discussion

### 4.1 Discussion

Findings suggest that the interaction of pain management clinic laws and Medicaid expansion could balance limiting inappropriate opioid prescribing while improving access to OUD treatment medications. We exploited the adoption of pain management clinic laws and Medicaid expansion using a generalized difference-in-difference framework. We found that states with a mixture of Medicaid and pain management clinic laws dispensed 9 fewer retail opioid prescriptions per 100 people (p<.01). We showed that this magnitude, 9 fewer retail opioid prescriptions per 100 people, is more prominent (in absolute terms) for states with just expansion of Medicaid or only adoption of pain management clinic laws. This reduction suggests that states with pain management clinic laws may have effectively reduced the over-supply of prescription opioids while still maintaining access to the prescription to effectively treat patients and manage chronic pain. We also used buprenorphine distributions as a proxy for OUD treatment and we found similar results. States with a mixture of Medicaid and pain management clinic laws had higher buprenorphine distributions with a magnitude of 0.5 kg per 100,000 population (p<.05). The effects were more substantial for states with a combination of Medicaid and pain management clinic laws than states that only had one of the two policies. Findings suggest that a policy mix can yield better results in reducing inappropriate prescriptions while increasing the availability of OUD treatment.

**Table A.1:** Impacts of Medicaid and pain management clinic laws on retail opioid prescriptions.

	Retail opioid prescriptions dispensed per 100 persons in the state population					
	(1)	(2)	(3)	(4)	(5)	(6)
Medicaid	1.235		1.307	1.327		2.675${}^{**}$
	(0.856)		(0.803)	(1.099)		(1.178)
Painmanagementclinic		$-6.808^{***}$	$-4.799^{***}$		$-2.991^{**}$	$-3.544^{***}$
		(1.007)	(0.955)		(1.421)	(1.348)
Painmanagementclinic×Medicaid			$-10.644^{***}$			$-6.240^{***}$
			(1.926)			(2.219)
State fixed effects	Yes	Yes	Yes	Yes	Yes	Yes
Year fixed effects	Yes	Yes	Yes	Yes	Yes	Yes
State specific trends	No	No	No	Linear	Linear	Linear
Controls	No	No	No	No	No	No
Cluster SE	State	State	State	State	State	State
HAC SE	Yes	Yes	Yes	Yes	Yes	Yes
Observations	612	612	612	612	612	612
$R^{2}$	0.971	0.975	0.978	0.988	0.988	0.989
Adjusted $R^{2}$	0.968	0.972	0.976	0.986	0.986	0.986

*Notes:* The analysis of retail opioid prescriptions dispensed per 100 persons in the state population uses publicly available data from the Centers for Disease Control and Prevention. Data on state oversight of pain management clinics is publicly available data from Rutkow et al. (2017). Data on state Medicaid expansion status was derived from the Kaiser Family Foundation. Data is a balanced panel for 50 U.S. states and the District of Columbia from 2006 to 2017 and comprises 612 observations. Standard errors are clustered at the state-level and robust to heteroskedasticity. Estimates are weighted with the state-level Medicaid beneficiary population. *** p<0.01, ** p<0.05, * p<0.1.

### 4.2 Limitations

Our study comes along with several limitations which warrant additional research. First, the CDC prescribing rates data from the IQVIA Xponent data set only include initial or refill prescriptions dispensed at retail pharmacies in the sample, paid for by commercial insurance, Medicaid, Medicare, or cash or its equivalent. Thus, we do not capture illegal opioids, but we also do not observe legal prescription opioids directed towards unlawful utilization. Second, we assume buprenorphine distributions in the ARCOS data are intended for the treatment of OUD. However, the ARCOS data does not identify buprenorphine products by formulation names or include patient diagnoses or indications; thus, we could not determine whether buprenorphine was indicated for pain. Third, we did not study methadone, another preferred medication for OUD treatment. We also do not account for whether state Medicaid programs allowed coverage of methadone, which may also impact buprenorphine availability. Fourth, we are unable to explain the underlying causal mechanisms that may explain study results. For example, it is of note that pain management clinic regulations were often a response to extreme cases of uncontrolled prescribing and enacted in areas that perhaps had more lax regulations to begin with. Similarly, we cannot ascertain whether areas with higher distributions of buprenorphine in states with pain management clinic regulations is in response to more utilization of buprenorphine for pain control versus opioid use disorder. Finally, future studies are needed to understand how opioid supply policies and Medicaid expansion might impact the availability of other OUD treatment medications.

### 4.3 Conclusion

Medicaid expansion and pain management clinic regulations resulted in fewer opioid prescriptions. Our results also showed higher buprenorphine prescriptions in states with both policies than states that did not enact both policies over the observation period. These findings indicate that policymakers might consider a mixed approach to opioid strategies that target the inappropriate supply of opioid prescriptions to help prevent the onset of OUD and simultaneously better meet the demand for OUD treatment.

**Table A.2:** Impacts of Medicaid and pain management clinic laws on buprenorphine distributions.

	Buprenorphine Sales, kilograms per 100,000 persons in the state population					
	(1)	(2)	(3)	(4)	(5)	(6)
Medicaid	0.027		−0.029	−0.014		$-0.042^{***}$
	(0.050)		(0.044)	(0.015)		(0.014)
Painmanagementclinic		0.243${}^{***}$	0.126${}^{**}$		0.021	0.031${}^{*}$
		(0.072)	(0.059)		(0.019)	(0.017)
Painmanagementclinic×Medicaid			0.724${}^{***}$			0.166${}^{***}$
			(0.102)			(0.049)
State fixed effects	Yes	Yes	Yes	Yes	Yes	Yes
Year fixed effects	Yes	Yes	Yes	Yes	Yes	Yes
State specific trends	No	No	No	Linear	Linear	Linear
Controls	No	No	No	No	No	No
Cluster SE	State	State	State	State	State	State
HAC SE	Yes	Yes	Yes	Yes	Yes	Yes
Observations	612	612	612	612	612	612
$R^{2}$	0.857	0.866	0.893	0.993	0.993	0.993
Adjusted $R^{2}$	0.841	0.850	0.880	0.991	0.991	0.992

*Notes:* The analysis of buprenorphine distributions in kilograms per 100,000 uses publicly available data from the Automated Reports and Consolidated Ordering System (ARCOS), available from the U.S. Department Of Justice, Drug Enforcement Administration, Diversion Control Division. State population data were drawn from the University of Kentucky Center for Poverty Research’s National Welfare Data. Data on state Medicaid expansion status was derived from the Kaiser Family Foundation. Data is a balanced panel for 50 U.S. states and the District of Columbia from 2006 to 2017 and comprises 612 observations. Standard errors are clustered at the state-level and robust to heteroskedasticity. Estimates are weighted with the state-level Medicaid beneficiary population. *** p<0.01, ** p<0.05, * p<0.1.

## Credit Author Statement

Dr. Shishir Shakya contributed in the project conceptualization, data curation, formal analysis, and writing the original draft of the manuscript.

Dr. Samantha Harris contributed in project administration and reviewing and editing the manuscript.

Each author has approved the final article for submission in Drug and Alcohol Dependence Reports.

## Declaration of Competing Interest

The authors declare that they have no known competing financial interests or personal relationships that could have appeared to influence the work reported in this paper.

## Appendix B Supplementary materials

Supplementary Data S1 Supplementary Raw Research Data. This is open data under the CC BY license http://creativecommons.org/licenses/by/4.0/ Supplementary Data S1
